# Controlled release evaluation of paracetamol loaded amine functionalized mesoporous silica KCC1 compared to microcrystalline cellulose based tablets

**DOI:** 10.1038/s41598-020-79983-8

**Published:** 2021-01-12

**Authors:** Marieh Pishnamazi, Hamid Hafizi, Mahboubeh Pishnamazi, Azam Marjani, Saeed Shirazian, Gavin M. Walker

**Affiliations:** 1 Department of Chemistry, Arak Branch, Islamic Azad University, Arak, Iran; 2 Department of Chemical Sciences, Bernal Institute, Synthesis and Solid-State Pharmaceutical Centre (SSPC), University of Limerick, Limerick, Ireland; 3 Institute of Research and Development, Duy Tan University, Da Nang, 550000 Vietnam; 4 The Faculty of Pharmacy, Duy Tan University, Da Nang, 550000 Vietnam; 5 Department for Management of Science and Technology Development, Ton Duc Thang University, Ho Chi Minh City, Vietnam; 6 Faculty of Applied Sciences, Ton Duc Thang University, Ho Chi Minh City, Vietnam; 7 The Faculty of Environmental and Chemical Engineering, Duy Tan University, Da Nang, 550000 Vietnam; 8 Laboratory of Computational Modeling of Drugs, South Ural State University, Chelyabinsk, 454080 Russian Federation

**Keywords:** Chemical engineering, Drug regulation, Chemical modification

## Abstract

In the pharmaceutical manufacturing, drug release behavior development is remained as one of the main challenges to improve the drug effectiveness. Recently, more focus has been done on using mesoporous silica materials as drug carriers for prolonged and superior control of drug release in human body. In this study, release behavior of paracetamol is developed using drug-loaded KCC-1-NH_2_ mesoporous silica, based on direct compaction method for preparation of tablets. The purpose of this study is to investigate the utilizing of pure KCC-1 mesoporous silica (KCC-1) and amino functionalized KCC-1 (KCC-1-NH_2_) as drug carriers in oral solid dosage formulations compared to common excipient, microcrystalline cellulose (MCC), to improve the control of drug release rate by manipulating surface chemistry of the carrier. Different formulations of KCC-1 and KCC-NH_2_ are designed to investigate the effect of functionalized mesoporous silica as carrier on drug controlled-release rate. The results displayed the remarkable effect of KCC-1-NH_2_ on drug controlled-release in comparison with the formulation containing pure KCC-1 and formulation including MCC as reference materials. The pure KCC-1 and KCC-1-NH_2_ are characterized using different evaluation methods such as FTIR, SEM, TEM and N_2_ adsorption analysis.

## Introduction

Recently, mesoporous silica materials as drug carrier^1–3^ have been widely used for improving the release rate of poorly water soluble drugs and delivering of therapeutic proteins^4^ due to their intrinsic properties^5^ such as non-toxic nature, good biocompatibility, easy functionalization, wide surface area with tunable pore size, high capacity of drug loading^6–13^, and superior capability to immobilize therapeutic molecules^14^. The majority of new drugs illustrate very poor bioavailability and solubility^15–17^, which lead to very low release rate during dissolution tests^18–23^. So far, in order to overcome this obstacle as one of the biggest challenges in pharmaceutical industry, different research groups have focused on displaying the potential of surface-functionalized mesoporous silica materials^24–26^ as an important host for oral drug controlled delivery systems^27–32^. Different researches have been done on enhancement and control of drug release rate and solubility^33^ by loading drug into mesoporous silica materials^19,34–37^.

Various studies have been carried out on drug administration methods and the influence of that on drug delivery systems^38^ in the human body^39–43^. Drug delivery systems play a key role to improve drug effectiveness and safety through controlling the drug release rate, release time and place in human body^44^. It is essential to develop a drug delivery system for all therapeutics to improve their influence on patients^45^. Paracetamol is a type of nonsteroidal anti-inflammatory drug^46^, that is used commonly as pain relief treatment. Paracetamol absorption is very fast in the small intestine which can cause severe side effects such as liver damage^47,48^. Then, it is essential to develop a controlled-release system for paracetamol^49^ especially in situation of overdose to overcome the problem of fatal liver damage^50^. Furthermore, the therapeutic behavior of paracetamol is improved by developing an engineered release system for that^51–53^.

Lim et al. investigated the release kinetics of ibuprofen and paracetamol utilizing fluorescent MSNs as drug carrier^12^. They found out that the release rate of paracetamol was higher than ibuprofen due to higher solubility of paracetamol. They also investigated the kinetics of drug release rate, and illustrated that ibuprofen release rate is dependent on solution pH, while higher release rate was obtained at lower pH. On the other hand, for the case of paracetamol, their results did not show significant difference at different pH and it was influenced by interaction between materials and molecules^12^. In another study, Szegedi et al. synthesized and amine-functionalized silica MCM-41 and SBA-15 using post-synthesise method. They studied the adsorption and release behaviour of ibuprofen from functionalized and drug loaded mesoporous silica. Their results revealed the possibility of ibuprofen controlled release rate by amine-functionalization of mesoporous silica^54^. AbouAitah et al. studied the release behaviour of Quercetin using amine-functionalized silica MCM-41 and KCC-1 as drug carrier. Their results illustrated that the synthesized MCM-41 and KCC-1 materials can be used as efficient drug controlled release host for long-term release in drug delivery systems^55^.

A series of mesoporous silica nanoparticles (MSNs) were used by Zhang et al. to develop and improve the solubility and release rate of poorly water drugs by increasing the capacity of drug loading on MSNs applying acetic acid as loading solvent via soaking method^56^. Their findings revealed that the capacity of drug loading is strongly associated to the pore size and pore volume of MSNs. Moreover, in another research, Qu et al. investigated the controlled release of Captopril utilizing mesoporous silica nanoparticles via different types of surfactants^57^. They explained that the pore size and morphology of mesoporous silica showed significant effect on drug release behavior and be able to control the release rate. Horcajada and co-authors tested MCM-41 mesoporous silica to study the effect of pore size of materials on drug delivery behavior^58^, which showed, reduction in the material pore size, results in a reduction in the drug delivery rate. Mellaerts et al. evaluated the bioavailability of poorly soluble drug (itraconazole) using drug loaded ordered mesoporous silica (OMS)^59^. They studied the in vivo and in vitro drug release rate from OMS and then compared with the release of pure crystalline drug. Their results showed that, OMS drug loaded displays faster drug release rate than pure itraconazole. Additionally, they compared the drug release rate from OMS with the sporanox as marketed drug and found that OMS can be applied as a promising drug carrier to improve the bioavailability of poorly water soluble drugs^59^.

Heikkila et al. loaded ibuprofen into mesoporous TUD-1 using soaking method to study the drug delivery system^60^. Their results indicated successful inclusion of ibuprofen with very high efficiency as model drug into the TUD-1 followed by significant potential of mesoporous silica materials to increase the drug release rate with 96% release after 2 h via dissolution test^60^. Ayad et al. utilized amine-functionalized mesoporous silica KIT-6 as a drug delivery carrier to study the controlled release behavior of drug^61^. They investigated the release behavior of drug at two different pH, gastric fluid (pH 2) and intestinal fluid (pH 7.4). They illustrated the significant influence of KIT-6-NH_2_ as mesoporous silica for drug loading to enhance drug release rate due to the interaction of hydrogen bonds of functional groups in the drug molecules, such as carboxyl and carbonyl groups^61^. Kinnari et al. studied and compared the effect of mesoporous silica and non-ordered mesoporous silica as drug carriers on the drug release behavior^27^.

Follmann et al. studied the sustained delivery of antitumor drug, camptothecin, which is poorly-water soluble drug using functionalized nanoporous silica KCC-1 with methyl groups. Then, functionalized KCC-1 was mixed with poly vinyl alcohol (PVA) and poly acrylic acid (PAA) to make hybrid aerogels to improve the drug delivery system^62^.

Mesoporous silica KCC-1^63,64^ with privilege morphology such as fibrous surface, numerous surface area and dendrimeric structure and in addition, superior mechanical stability can be considered as one of the unprecedent types of mesoporous silica in drug delivery systems as drug carrier^65^.

Herein, first, we illustrated the synthesis of mesoporous silica^66^ KCC-1 which possesses high surface area due to existence of dendrimeric^67^ fibrous structure and their respective channels, which make it as an attractive host for drug delivery^68–76^. It should be pointed out that, according to the literature, the fibrous morphology of KCC-1 silica is unprecedented, thereby it is considered as a high-performance carrier for therapeutic molecules. KCC-1 demonstrated supreme physical properties such as high surface area, fibrous morphology, and great thermal/hydrothermal as well as mechanical stability. The singularity of KCC-1 is related to its high surface area owing to fibrous nature of KCC-1 instead of pores, which causes to be easily available^77^. Then, we presented the utilization of KCC-1-NH_2_ as drug carrier to improve the drug release behavior during the in vitro dissolution tests. The functionalized KCC-1 and the pure KCC-1 were characterized using different characterization techniques such as scanning electron microscopy (SEM), transmission electron microscopy (TEM), Fourier transform infrared spectroscopy (FTIR), and Nitrogen adsorption/desorption. Paracetamol^78^ was used as model drug in this project.

## Materials

Cetyltrimethylammonium bromide (CTAB) (Merck KGaA, Germany) was purchased from Merck. 3-aminopropyl triethoxysilane (APTS), Tetraethylorthosilicate (TEOS, 98%), toluene anhydrous, 1-pentanol, urea and cyclohexane were purchased from Sigma-Aldrich. Paracetamol (4-acetamidophenol, Phion) was used as model drug in all formulations. Microcrystalline cellulose (MCC SANAQ 101 L USP/NF/EP) was used as excipient in preparation of tablets.

## Methods

### Synthesis of mesoporous silica KCC-1

Purely dendritic fibrous siliceous KCC-1 was synthesized according to a method by Polshettiwar and co-workers^68^. In brief, first a suspension of 1 g CTAB and 0.6 g of urea in 30 ml deionized water was prepared. Afterward, the suspension was stirred for 2 h at room temperature. Then, the second suspension was prepared using 30 ml of cyclohexane, 1.5 ml of 1-pentanol and 2.5 g of tetraethyl orthosilicate (TEOS). The first prepared suspension was added to the mixture under stirring. The stirring of the final suspension continued for further 2 h. Then, the mixture was transferred to a Teflon-sealed hydrothermal reactor and, then placed in oven for 5 h at 120 °C. As next step, the suspension was centrifuged in 6500 rpm for 3 min. Then, the solid product was washed twice with acetone and deionized water. The final materials dried in oven for 24 h at 60 °C. Finally, the white powder was furnace-calcined at 550 °C for 5 h in order to remove CTAB template (calcination process).

### Functionalization of mesoporous silica KCC-1 using aminopropyl triethoxysilane (APTES)

For functionalization of the KCC-1 with n-propyl amine groups (Pr-NH_2_) to obtain KCC-1-NH_2_, post grafting method was used^79^. For achieving this goal, 1 g of KCC-1 was added to 50 ml of dry toluene and ultrasonicated for 15 min. Then, 1 ml of APTES was added to the mixture and magnetically stirred under reflux at 110 °C for 12 h. After cooling down to room temperature, the mixture was filtered and washed several times with toluene and ethanol and oven-dried at 60 °C for 24 h. The white powder of KCC-1-NH_2_ was then successfully obtained.

### Drug loading procedure

For paracetamol loading into the KCC-1 and KCC-1-NH_2_, solvent evaporation method was employed. 150 mg of KCC-1 and 150 mg of paracetamol were mixed, and 1 ml of ethanol was added to the mixture. As the next step, the mixture was ultrasonicated for 10 min and dried in the room temperature for 48 h^19^. This procedure was repeated for drug loading on KCC-1-NH_2_ as well.

### Tableting procedure

A single-punch tablet press (Gamlen Tableting GTD-1 D series) was used to compact the 100 mg drug loaded KCC-1 and KCC-1-NH_2_ to tablet shape, in 6 mm die by direct compaction method. 400 kg was considered as tablet loading, and the compaction rate was set at 180 mm/min^80,81^.

### Dissolution test procedure

In vitro dissolution test was done utilizing a pharma PTWS 120D 6-station tablet dissolution testing instrument (Hainburg, Germany). Phosphate buffer was used as dissolution medium at 37 ± 0.5 °C^80,82,83^. In order to study the release characteristics of paracetamol from the parent mesoporous silica KCC-1 and KCC-1-NH_2_^84,85^, in vitro release experiments were done by keeping constant the stirring rate at 50 rpm and temperature at 37 ± 0.5 °CC for paracetamol according to the USP^64^. Dissolution tests were done with 900 ml of buffer solution as medium during 4 h for phosphate buffer solution (PBS) at pH 7 and gastric buffer solution at pH of 1.2. During the dissolution test, 5 ml of each sample was withdrawn every 10 min until 60 min, then every 1 h. The buffer was replaced immediately with the same amount to keep the total volume constant at 900 ml. At the end, all samples were diluted using the same buffer and analyzed utilizing UV at wavelength of 249 nm.

### Scanning electron microscopy and transmission electron microscopy

For analysing the morphology and particle size of KCC-1 and KCC-1-NH_2_ samples, field-emission electron microscope (FE-SEM, Hitachi SU8030, Japan) was carried out. The surface morphology of KCC-1 and KCC-1-NH_2_ were characterized utilizing high-resolution transmission electron microscopy (HRTEM, JEOL JEM-2011F electron microscope). Sample preparation was done by drop-casting a suspension of sample in ethanol on TEM Cu grids followed by drying in air.

### Fourier transform infrared spectroscopy

Fourier transform infrared (FTIR) spectroscopy was performed for the functionalized and non-functionalized KCC-1 samples. Nicolet Nexus FTIR spectrometer between 400 and 4000 cm^−1^ was utilised and equipped with an attenuated total reflectance accessory (ATR).

### N_2_ adsorption–desorption isotherms

To study the Brunauer–Emmet–Teller equation (BET) specific surface area of KCC-1 and KCC-1-NH_2_ samples, the multipoint method was applied. For measuring the pore size distribution of the samples, Barrett–Joyner–Halenda (BJH) method on a Quantachrome Autosorb-1 instruments, using the desorption branches of the isotherms was considered. The samples were vacuum degassed at 120 °C overnight before starting the analysis.

## Results and discussion

### SEM and TEM analysis

**Figure 1:**
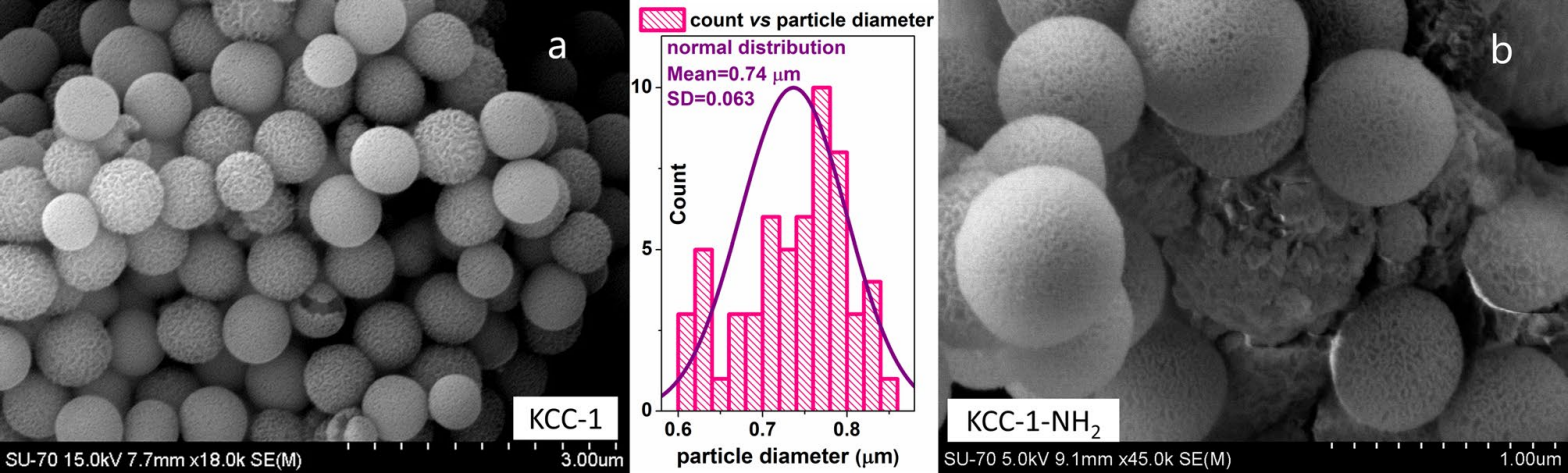
FESEM images of (**a**) KCC-1 and (**b**) KCC-1-NH_2_. The middle diagram is particle size distribution for KCC-1.

For analyzing the particle size, structural and morphological features of KCC-1 and KCC-1-NH_2_, SEM and TEM analyses were carried out. From SEM images in Fig. 1, it is observed that both particles have spherical morphology, which are made by nano-sized dendrimeric fibers. The SEM observations of samples show that the morphology and size do not change during functionalization process by NH_2_ groups. Also, according to the particle size distribution histogram of KCC-1, the mean particle size diameter of KCC-1 prepared in this work is about 0.74 μm. Also, no considerable changes were observed in KCC-1 shape and particle size distribution after functionalization using amine groups^86^.

**Figure 2:**
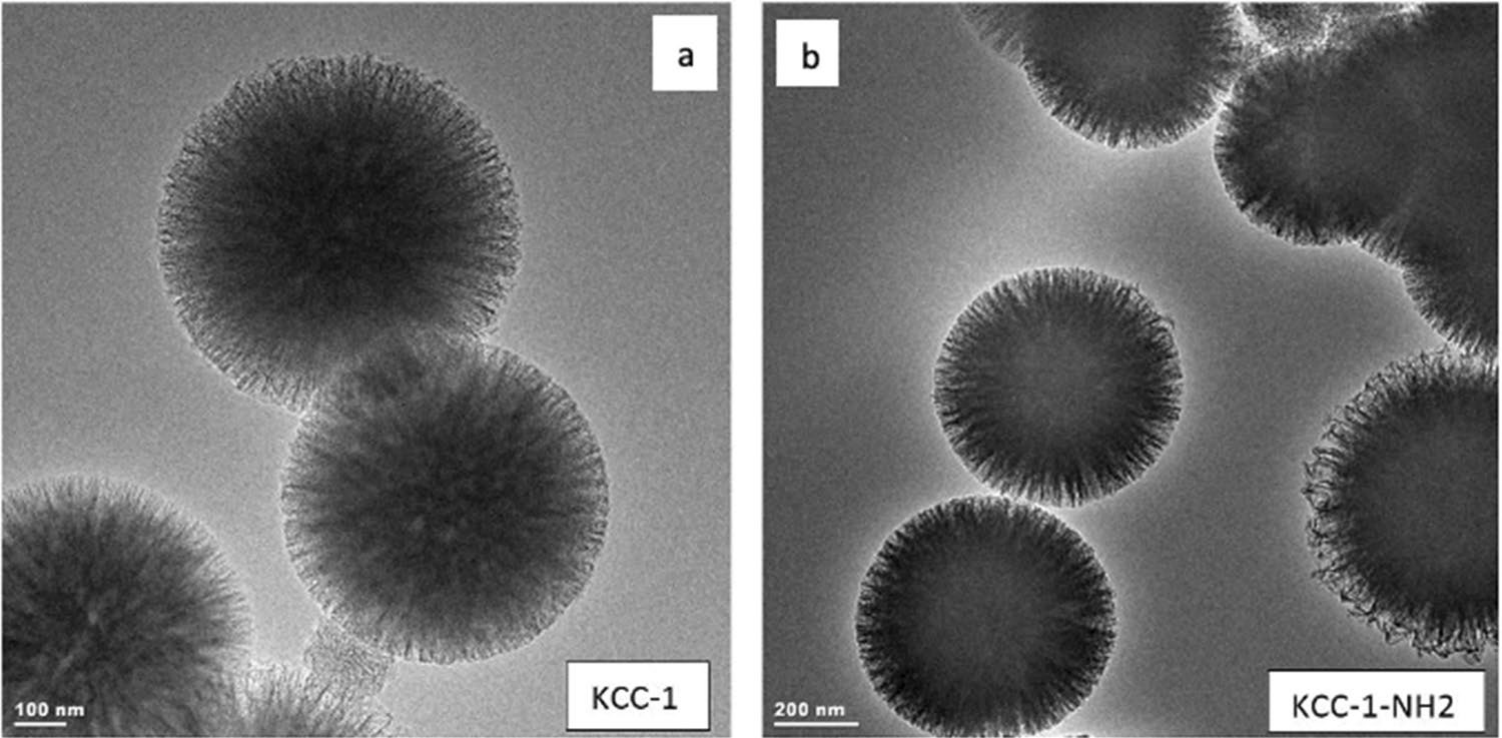
TEM images of (**a**) KCC-1 and (**b**) KCC-1-NH_2_ particles.

To investigate the structure of KCC-1 and KCC-1-NH_2_, TEM analysis was performed. In Fig. 2, the TEM images reveal that both pure KCC-1 and KCC-1-NH_2_ possess monodispersed fibrous silica spheres where the dendrimeric fibers uniformly grow—along with the free radial directions—from the center of the spheres. Also, after surface functionalization, there was no noticeable change in the fibrous and spherical structure of the KCC-1, and the structure remains intact. It is reported that the existence of nanoporous silica fibers in the structure of KCC-1 efficiently renders molecular accessibility to the surface of KCC-1 in comparison to other types of mesoporous silica materials which makes KCC-1 as an attractive candidate for drug delivery and controlled release applications^49,87,88^.

### FT-IR analysis

**Figure 3:**
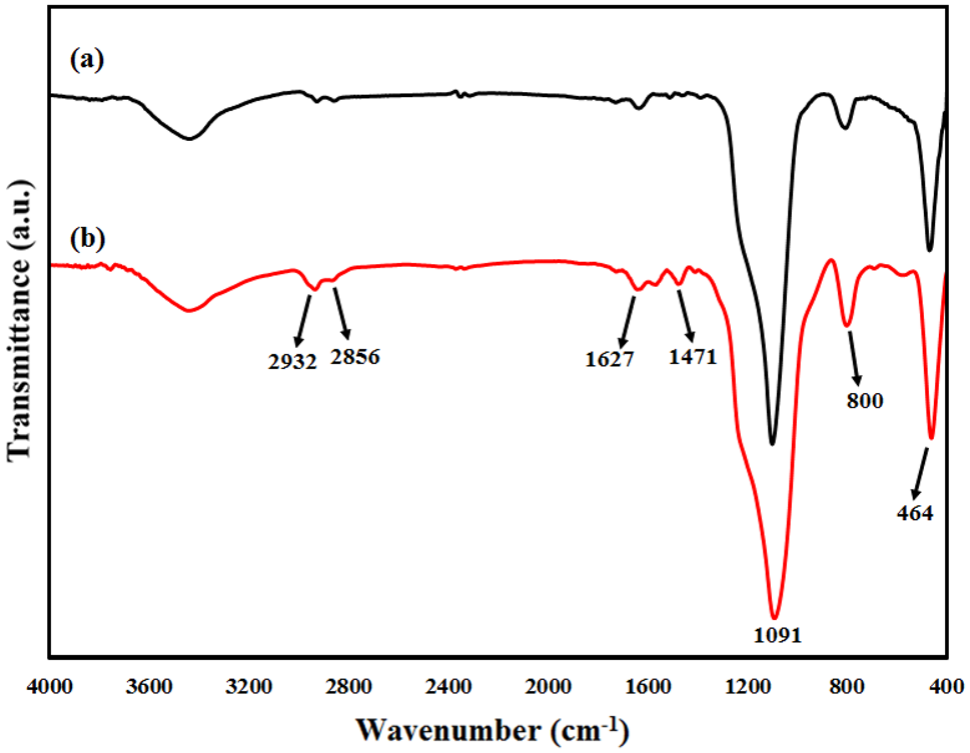
FTIR spectra of (**a**) KCC-1 and (**b**) KCC-1-NH_2_ samples.

Figure 3 illustrates the FT infrared spectra of the KCC-1 (curve a) and the KCC-1-NH_2_ (curve b) recorded in the region 4000–400 cm^−1^. For both samples, the absorption peaks at 1091 cm^–1^, 800 cm^–1^, and 464 cm^–1^ were assigned to asymmetric, symmetrical stretching and bending vibration of Si–O–Si bond, respectively. The spectral band at 1627 cm^–1^ in samples is due to the –OH deformation band of water molecules remained in the matrix. For the KCC-1-NH_2_ sample, the band at 1471 cm^–1^ in the spectrum indicated the existence of the N–H bending vibration of the –NH_2_ groups. Two bands at 2932 cm^–1^ and 2856 cm^–1^ are corresponded to the ν_CH_ of the –CH_2_ groups of propyl chain, indicating the successful formation of amine groups after modification.

### N_2_ adsorption–desorption isotherms

**Figure 4:**
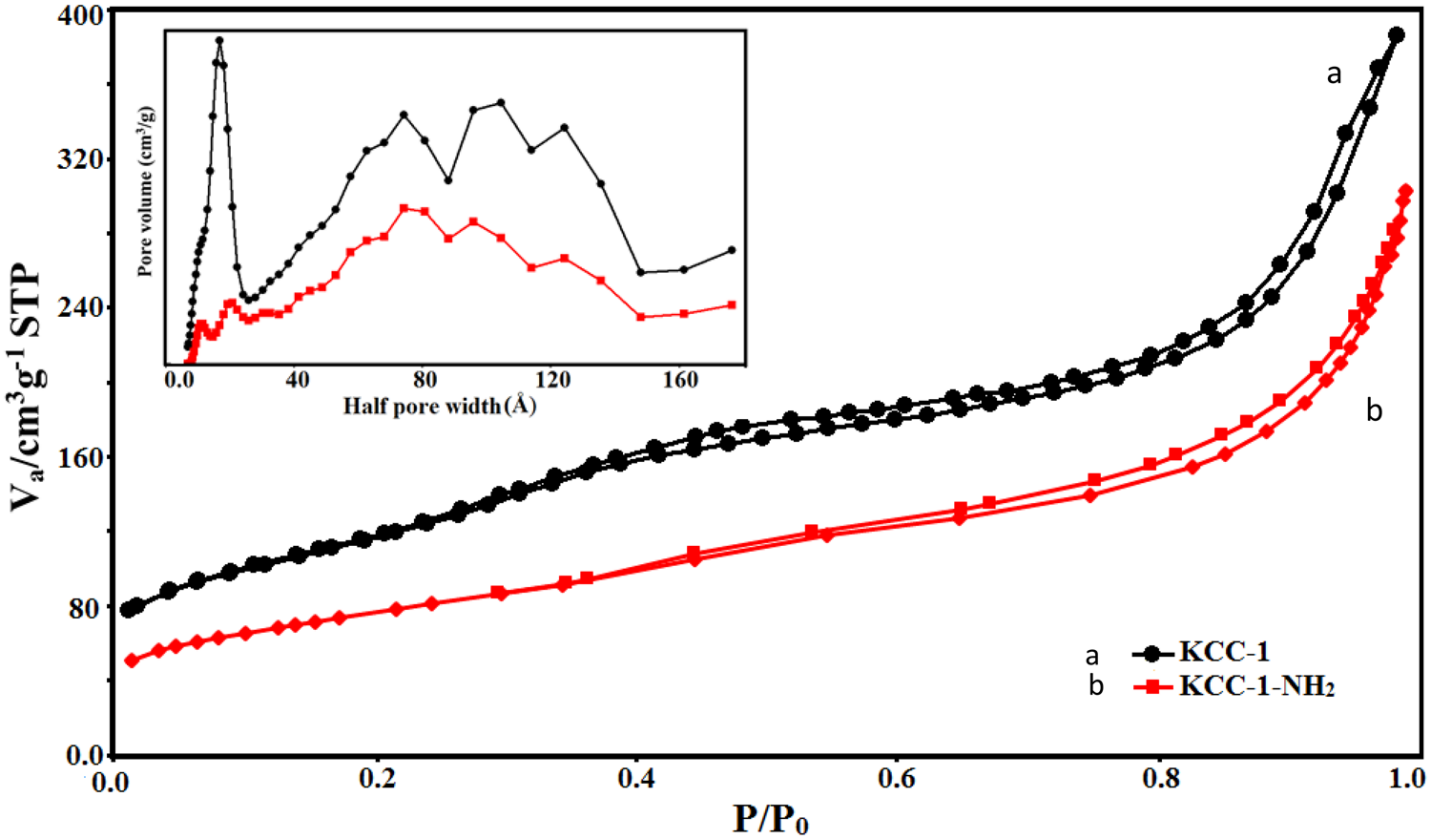
Nitrogen adsorption–desorption of KCC-1 and KCC-1-NH_2_ and pore size distribution (inset) of samples utilizing non-local density functional theory (NLDFT) model.

N_2_ adsorption–desorption analysis was performed for study of the porous structural feature of the samples. Figure 4 shows the results of obtained N_2_ adsorption–desorption isotherms for KCC-1 and KCC-1-NH_2_ at 77 K and *P*/*P*_0_ = 0.99. Both curves have type- IV isotherm based on the IUPAC classification, which is usual for mesosphere silica^55^. As it is clear in the Fig. 4 and Table 1, the KCC-1 sample illustrated wider pore size distribution in comparison with KCC-1-NH_2_. In addition, KCC-1 shows higher specific surface area compared to the KCC-1-NH_2_. Pore size distribution of samples using non-local density functional theory (NLDFT) model are also shown in Fig. 4. The average pore diameter of KCC-1 and KCC-1-NH_2_, according to the BJH method, is 2.4 and 2.2 nm, respectively. There is a reduction in the specific surface area and pore size distribution. All these changes can prove a successful functionalization process. These—a reduction in surface area, pore size and pore volume- are typical phenomena observed during functionalization of mesoporous silica materials via post-modification or post-grafting method^89^.

**Table 1:** Textural properties of mesoporous silica KCC-1 and KCC-1-NH_2_ according to the BET and BJH models.

Samples	*S*_BET_ (m^2 ^g^−1^)	*V*tot. (cm^3^ g^−1^)	*D* (nm)
KCC-1	356	0.49	2.4
KCC-1-NH_2_	248	0.40	2.2

*S*_BET_ specific surface area according to the BET method, *V*_tot._ total pore volume, *D* BJH average pore diameter.

### Thermogravimetric analysis (TGA)

**Figure 5:**
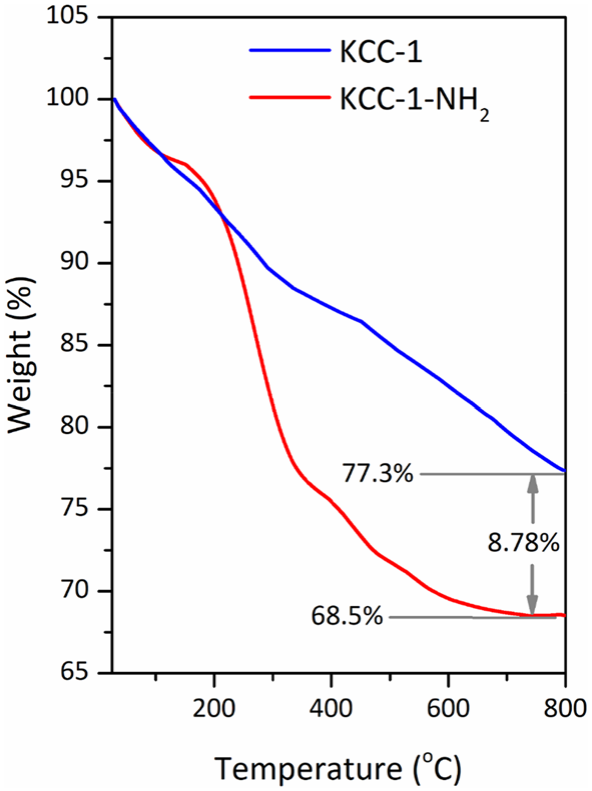
TGA curves of KCC-1 and KCC-1-NH_2_ under N_2_ atmosphere from 25 to 800 °C at a heating rate of 10 °C/min^−1^.

Thermal stability of the KCC-1 and KCC-1-NH_2_ was studied by TGA analysis^90^ under a nitrogen atmosphere. As indicated in Fig. 5, a weight-loss below 120 °C was seen for the samples due to the loss of organic solvent and/or physiosorbed water^91^—which were used during the preparation of KCC-1 and KCC-1-NH_2_—from the surface of the samples. The weight loss of KCC-1 between 120 and 800 °C was mostly because of the thermal decomposition of silanol hydroxyl groups (Si–OH) and residual organic moiety (CTAB). Under the same condition, the weight of grafted APTES on the fibers of KCC-1 determined from the TGA was about 8.8%. For KCC-1-NH_2_, the weight loss between 400 and 800 °C is mainly because of the decomposition of the organic residue [i.e., organic part (amino propyl) of the covalently attached APTES], beside the above-mentioned weight loss for pure KCC-1. TGA results proved that a large amount of the APTES molecules (8.8%) have been chemically attached onto the surface of silica fibers of KCC-1 and these samples are stable up to 450 °C (> 30% weight loss) in a nitrogen flow atmosphere.

### X-ray diffraction

**Figure 6:**
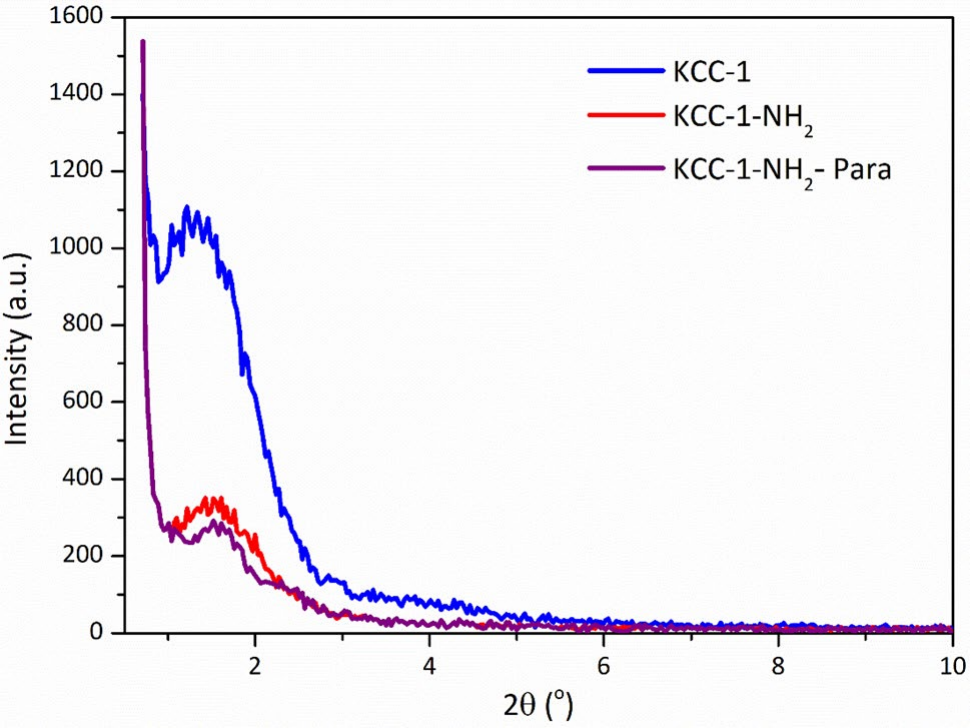
Low angle-XRD patterns of the samples.

To understand the crystalline structure of the KCC-1, KCC-1-NH_2_, and KCC-1-NH_2_-Pra, low-angle X-ray diffraction (L-XRD) patterns of all materials were recorded on a *PANalytical Empyrean* diffractometer, operated at 40 kV and 40 mA using Cu Kα radiation (λ = 0.154 nm). The patterns of the samples are shown in Fig. 6. This silica materials demonstrated only a broad diffraction peak at 2*θ* = 1°–2.5°, indicating the low ordering of the silica framework in samples. This kind of broad diffraction peak at low angle has already been interpreted in terms of disordered pore structure and wormhole-motif structure in previous studies—where functionalized materials were prepared by post-grafting method^57–61^. The peak intensity for pure mesoporous KCC-1 was somewhat decreased upon surface functionalization with APTES, indicating that the introduction of silane coupling agent affected the pore structure. After drug loading in KCC-1-NH_2_ (KCC-1-NH_2_-Para sample) the peak intensity was decreased slightly again, which was predictable.

### In vitro dissolution tests of drug loaded KCC-1 and KCC-1-NH_2_

Three different formulations are considered to investigate the effect of KCC-1 and KCC-1-NH_2_ on paracetamol release rate. The first formulation was considered as reference and contains MCC as a common excipient in oral solid dosage formulation and paracetamol as drug, 1:1 (w/w). The second formulation includes drug loaded KCC-1 as drug carrier, 1:1 (w/w). In addition, in the third formulation, drug loaded KCC-1-NH_2_ is utilized as drug carrier, 1:1 (w/w). The formulation containing MCC was considered to compare the drug release rate from the common oral solid formulation and mesoporous silica-based drug loaded of oral solid dosage formulation.

**Figure 7:**
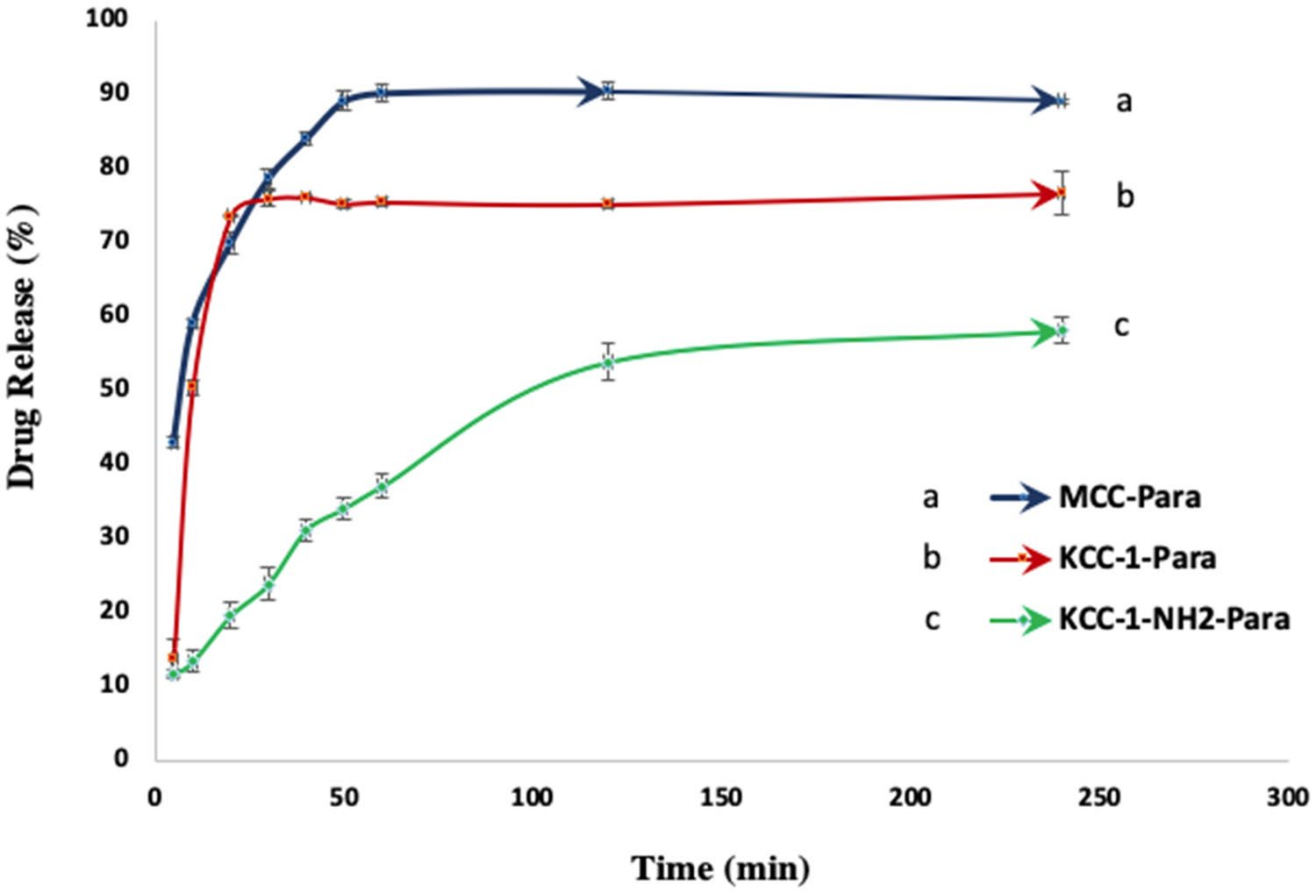
Release rate of paracetamol from MCC-Para, KCC-1-Para and KCC-1-NH_2_-Para tablets at pH 7.

**Figure 8:**
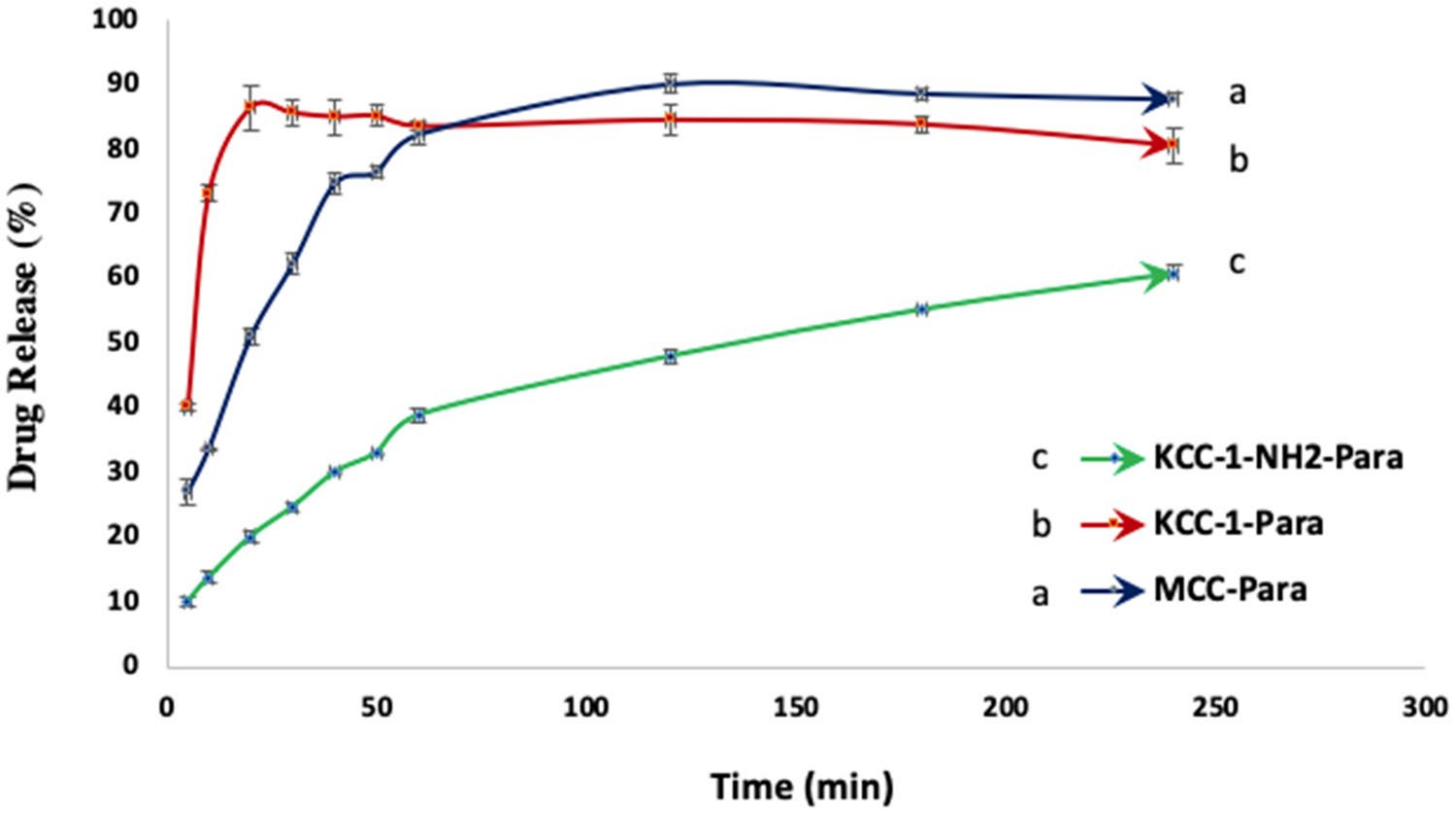
Release rate of paracetamol from MCC-Para, KCC-1-Para and KCC-1-NH_2_-Para tablet at pH 1.2.

The release profiles of those three formulations are plotted in Figs. 7 and 8. As presented in the graphs, the concentration of released paracetamol at both pH, 1.2 and 7, is a function of time and monitored using UV analysis. The graphs also illustrate the ability of mesoporous silica KCC-1 and KCC-1-NH_2_ to use as drug carrier for regulating the drug release rate compared to MCC as a common excipient in oral solid dosage formulations. In addition, the results show the reduction of drug release rate for KCC-1-NH_2_ and its ability to use as drug carrier for prolonged and controlled release of drug. Figures 7 and 8 reveal the cumulative percent of released paracetamol from MCC-Para (paracetamol), KCC-1-Para and KCC-1-NH_2_-Para tablets at pH 7 for 240 min. The graphs show that the release rate of paracetamol from KCC-1-NH_2_-Para tablet is slower than KCC-1-Para tablet. In addition, in Fig. 8, it is clear that the paracetamol release equilibrium rates are higher for the KCC-1-Para tablet compared to the KCC-1-NH_2_-Para tablet at pH 1.2. The release rate can be dependent on the stronger and weaker interactions between amine-functional groups of KCC-1-NH_2_ and functional groups in paracetamol including amide (–CO–NH_2_) and hydroxyl (–OH) groups, respectively^23,54,92^. These interactions result in slower release rate for the KCC-1-NH_2_-Para. The release graphs presented that functionalized KCC-1 was able to adjust the drug release rate due to the existence of the –NH_2_ groups by reducing the invasion of PBS in the pores and leads to a lag in drug release^23^. Moreover, steric hindrance of amine groups leads to inhibiting the drug release from silica KCC-1^55,64^. Another reason for reduction of drug release rate could be due to narrower pore size of silica KCC-1-NH_2_ after amine functionalization^54,55^.

Moreover, the structure of mesoporous silica KCC-1 such as wide surface area, pores, fibrous and dendrimeric structure leads to enhance the drug loading capacity and improve the transmission rate of drug molecule by controlling the release rate^66^. However, the drug release from the mesoporous silica was controlled by diffusion mechanism through their pores. Therewith, drug release rate is dependent on the chemical structure of model drug and interaction between the paracetamol, functional decoration of mesoporous silica and phosphate buffer solution. Furthermore, the drug release is influenced by the existence of hydrophilic groups in the paracetamol structure such as hydroxyl, carboxyl and amide groups^13,23^. The drug release can be also related to the pore diameter of mesopore silica. Effect of pH on the release rate of paracetamol is not significant, but under pH 1.2, the release rate is slightly higher which could be due to protonation of hydroxyl groups in KCC-1 and amine groups in KCC-1-NH_2_^48,64^. Higher solubility of paracetamol in phosphate buffer could be due to the existence of hydroxyl, carboxyl and amide groups which are known as hydrophilic groups^12,92–95^.

## Conclusions

In this work, mesoporous silica KCC-1 was synthesized by sol–gel-hydrothermal method and functionalized by amino-groups using post grafting method to prepare KCC-1-NH_2_ for controlled release applications. Both synthesized and functionalized KCC-1 were characterized utilizing SEM, TEM, FTIR and N_2_ adsorption analysis in terms of pore size, surface area, morphology, structure and functional groups. Afterwards, release characteristics of drug loaded KCC-1 and KCC-1-NH_2_ were studied at different pH, 7 and 1.2. As the results presented, the produced mesoporous silica as drug carrier was suitable for drug loading due to its outstanding properties. The drug release rate was remarkably impressed by intermolecular interaction between paracetamol and KCC-1-NH_2_, and between paracetamol and phosphate buffer solution as well. Based on these findings, mesoporous KCC-1-NH_2_ can be considered as a promising carrier for drug delivery systems to control the drug release rate, specifically for highly soluble drugs, such as paracetamol.
